# Views about integrating smoking cessation treatment within psychological services for patients with common mental illness: A multi‐perspective qualitative study

**DOI:** 10.1111/hex.13182

**Published:** 2020-12-23

**Authors:** Gemma M. J. Taylor, Katherine Sawyer, David Kessler, Marcus R. Munafò, Paul Aveyard, Alison Shaw

**Affiliations:** ^1^ Addiction and Mental Health Group (AIM) Department of Psychology University of Bath Bath UK; ^2^ Centre for Academic Primary Care Bristol Medical School University of Bristol Bristol UK; ^3^ MRC Integrative Epidemiology Unit School of Psychological Science University of Bristol Bristol UK; ^4^ Nuffield Department of Primary Care Health Sciences University of Oxford Oxford UK

**Keywords:** anxiety, IAPT, depression, improving access to psychological therapies, primary health care, smoking cessation, tobacco smoking treatment

## Abstract

**Background:**

Tobacco smoking rates are significantly higher in people with common mental illness compared to those without. Smoking cessation treatment could be offered as part of usual outpatient psychological care, but currently is not.

**Objective:**

To understand patient and health care professionals' views about integrating smoking cessation treatment into outpatient psychological services for common mental illness.

**Design:**

Qualitative in‐depth interviews, with thematic analysis.

**Participants:**

Eleven Improving Access to Psychological Therapies (IAPT) psychological wellbeing practitioners (PWPs), six IAPT patients, and six stop smoking advisors were recruited from English smoking cessation, and IAPT services.

**Results:**

Patients reported psychological benefits from smoking, and also described smoking as a form of self‐harm. Stop smoking advisors displayed therapeutic pessimism and stigmatizing attitudes towards helping people with mental illness to quit smoking. PWPs have positive attitudes towards smoking cessation treatment for people with common mental illness. PWPs and patients accept evidence that smoking tobacco may harm mental health, and quitting might benefit mental health. PWPs report expertise in helping people with common mental illness to make behavioural changes in the face of mood disturbances and low motivation. PWPs felt confident in offering smoking cessation treatments to patients, but suggested a caseload reduction may be required to deliver smoking cessation support in IAPT.

**Conclusions:**

IAPT appears to be a natural environment for smoking cessation treatment. PWPs may need additional training, and a caseload reduction. Integration of smoking cessation treatment into IAPT services should be tested in a pilot and feasibility study.

**Patient or public contribution:**

Service users and members of the public were involved in study design and interpretation of data.

## 1 INTRODUCTION

Smoking tobacco is the world's leading cause of preventable illness and death.^1^ One in every two smokers will die of a smoking‐related disease, unless they quit.^2^, ^3^ Globally, smoking prevalence has decreased from 29% during the 1990s to about 15% in recent years.^4^ However, smoking rates in people with common mental illness, like depression and anxiety, are at least twice the rate of those without common mental illness. For example, in England in 2015 it was estimated that 34% of people with depression, and 29% of people with anxiety smoke.^5^ This population is more heavily addicted, suffer from worse withdrawal,^6^ and has a 19% reduction in the odds of achieving abstinence when making a quit attempt,^7^ but are as motivated to quit as the general population.^8^ These differences increase mortality in people with common mental illness when compared to the general population (mortality rate ratio, 1.92 (95% CI: 1.91 to 1.94).^9^

One major barrier to implementing smoking cessation treatments in this population is the widely held misconception that smoking tobacco offers mental health benefits, quitting may interfere with mental illness treatment, and that smoking should be addressed once mental health has improved.^10^ However, there is no clear reason why mental illness and tobacco addiction cannot be treated simultaneously, and no evidence to suggest that stopping smoking causes psychological harm.^11^ Conversely, there is growing evidence that smoking may worsen mental health through the tobacco withdrawal cycle,^12^ and that stopping smoking may improve mental health, an effect size equal to anti‐depressant treatment.^11^ One explanation for this is that when someone starts smoking, there are initial rewarding effects of tobacco on mood and cognition. However, as the person becomes used to the effects of tobacco, these reasons for smoking tend to diminish, as the alleviation of withdrawal symptoms such as low mood, irritability, poor concentration, restlessness and anxiety gain prominence.^13^, ^14^ People who smoke regularly will feel these withdrawal symptoms much of the time, with short periods of relief only while they smoke and shortly after. When caught in this cycle, people can mistakenly believe that smoking helps relieve symptoms of anxiety, depression, low mood or stress.^15^

In England, people with common mental illness are usually referred/self‐referred to local smoking cessation services for smoking cessation treatment. English smoking cessation services are commissioned to deliver evidence‐based smoking cessation treatments (eg nicotine replacement therapy or varenicline offered in combination with behavioural therapy),^16^ are usually based in primary care or in the community, and treatments are delivered by ‘stop smoking advisors’ who are trained by the National Centre for Smoking Cessation and Training (NCSCT).^17^ The NCSCT is a social enterprise that supports the National Health Service (NHS) and Local Authorities to deliver evidence‐based smoking cessation treatments, and their training programme is aligned with National Institute of Clinical Excellence (NICE) guidelines for smoking cessation.^16^, ^18^, ^19^ NCSCT and NICE emphasize the importance of supporting people with mental illness to stop smoking.

A Cochrane review of smoking cessation interventions for people with current and historical depression found that adding psychosocial mood management to usual smoking cessation treatment (eg nicotine replacement therapy) increased smoking cessation rates compared to usual smoking cessation treatment alone, risk ratio of 1.47 (95% CI: 1.13 to 1.92).^20^ Smoking cessation support could be appropriately placed in psychological services whereby people who want help to stop smoking would be offered the option of receiving integrated treatment for their mental illness and tobacco addiction; however, there is evidence that there could be potential systemic barriers to this approach.

A systematic review of mental health professionals’ attitudes to treating smoking cessation in people with mental illness included 38 studies involving 16 369 mental health professionals.^10^ What was clear from the review and other research in this area is that negative attitudes towards smoking cessation are widespread in inpatient settings where professionals work with patients who have severe mental illness (ie psychosis), and in institutions that operate using predominately medically based treatment models (ie hospitals).^10^, ^21^, ^22^ But what is not clear is whether or not these attitudes are widespread in institutions that work with patients with common mental illness, or in institutions that operate using predominately psychologically based treatment models (ie psychological therapies services).

In England, people with common mental illness are usually referred to a community based psychological therapies service, known as ‘Improving Access to Psychological Therapies (IAPT)’. In IAPT, patients receive cognitive behavioural therapies (CBT) to improve mood symptoms and quality of life. IAPT could deliver smoking cessation treatment alongside CBT, but currently does not. Therefore, the aim of this qualitative study was to understand whether it was possible to integrate smoking cessation treatment into IAPT usual care for people with common mental illness by understanding the relevant concerns of patients and staff.

### 1.1 Research objectives

The objectives of the study were to:

1. Understand patient experiences of comorbid smoking and common mental illness;
2. Understand patient views about treatments for tobacco addiction and common mental illness, including integrated treatment of both;
3. Understand health professional's knowledge and views of integrated treatment for tobacco addiction and common mental illness, including pharmacotherapy as an aid to smoking cessation;
4. Collect data to inform a potential smoking cessation intervention and training for integration into IAPT.

## 2 METHODS

The protocol for this study has been pre‐registered (https://osf.io/z7vsy/), the pre‐print is available via medRxiv (https://doi.org/10.1101/2020.02.18.20024596), and the anonymized data are available to researchers via application to the University of Bath (https://doi.org/10.15125/BATH‐00921). We have followed COREQ reporting guidelines in writing this manuscript.^23^ Ethics approval for this study was received from the NHS research ethics committee on 13 July 2017 (Reference 17/WM/0251).

We conducted semi‐structured interviews with IAPT psychological wellbeing practitioners (PWPs) and patients, and stop smoking advisors. Topic guides are provided in the supplement (Tables S1‐S3). Data are presented thematically rather than by interview question, if responses to each question are of interest to researchers the anonymized transcripts are available via the University of Bath's Research Data Archive (https://doi.org/10.15125/BATH‐00921).

### 2.1 Sampling and recruitment

Informed consent was obtained from all participants. We recruited participants from IAPT and smoking cessation services in England until we generated adequate information power.^24^ Information power is more suitable for pragmatic applied health research than is data saturation. ‘Data saturation’ was originally developed for grounded theory analysis.^25^ Information power indicates that the more information the sample holds relevant to the study, the lower number of participants is needed. We followed the guidance outlined by Malterud et al,^24^ and assessed sample size during analysis, based on this we agreed as a team that we reached sufficient information power. Information power was determined based on the aim of the study, sample specificity, use of established theory, quality of dialogue, and analysis strategy. Our aims were broad, the sample specificity was dense, our theory was applied, the dialogue was strong, and we conducted analysis at the case‐ and cross‐case‐levels.

PWPs and stop smoking advisors were recruited using a snowballing strategy at the service‐level. Using a purposive approach we aimed to interview males and females, and those who were less (≥1 to <2 years) and more experienced (≥2 years) practitioners. IAPT patients were recruited by PWPs during appointments. We aimed to interview patients with different types of mental illness deemed treatable in IAPT.

#### 2.1.1 Eligibility criteria

We included adults aged ≥18‐years. PWPs were included if they were employed by IAPT services, and were non‐ or ex‐smokers. Stop smoking advisors were included if they were employed by smoking cessation services and were NCSCT‐trained. IAPT patients were included if they had a common mental illness and were currently receiving IAPT treatment or completed IAPT treatment within 1 year of the interview, and smoked daily for ≥1 year.

Participants were not paid for their contribution but were reimbursed for travel to the interview venue.

### 2.2 Data collection

Interviews were conducted between September 2017 and April 2018, in‐person at the University of Bristol or by telephone. Interviews were audio recorded and lasted typically 60 minutes.

Topic guides were used to assist questioning during interviews with flexibility to reflect emergent findings. The interviewer (*GT*) used open‐ended questioning to elicit participants’ experiences and views, and participants were asked to provide examples to avoid reliance on ‘hypothetical’ accounts.

Data were transcribed by a third‐party service. A researcher did a 50% check of audio data against the transcripts. Transcripts were not checked by participants.

### 2.3 Data analysis

*KS* and *GT* led the analysis with support from *AS*. Researchers held a critical realist perspective, and data were analysed using a framework approach to thematic analysis, following Braun and Clarke's method^26^; this allows for anticipated themes (ie deductive coding) and emergent themes (ie inductive coding), provides a systematic model for managing and mapping the data, and is suitable for comparisons within and between cases, as well as overriding themes.^27^, ^28^

For PWPs we used the ‘theoretical domains framework’ (TDF) to deductively identify implementation barriers and facilitators.^29^ For IAPT patients we deductively used the ‘capability, opportunity, and motivation—behaviour change model’ (COM‐B) that is designed for characterizing and designing behaviour change interventions.^30^ Inductive codes were data‐driven, and remained close to participants language where possible.

GT, KS and AS read each transcript, and listened to audio recordings before coding transcripts. Data were coded in four phases:

1. *GT, KS* and *AS* started with inductive line‐by‐line coding.
2. After coding three transcripts, *GT, KS* and *AS* compared labels and agreed a set of codes to apply to all subsequent transcripts. Then codes were grouped into categories providing a working analytical framework.
3. Deductively coded concepts from the TDF and COM‐B models were applied to the data where appropriate; some data were coded both inductively and deductively (Table S4).
4. Overarching themes and subthemes were developed based on what was necessary for intervention development and implementation.

N‐Vivo software was used to apply the working analytical framework for phases 1 and 2. For phases 3 and 4 we used Microsoft Word and Excel.

### 2.4 Patient and public involvement

The research aims and design were reviewed by the UK Centre for Tobacco and Alcohol Studies Smokers’ Panel. In general, the study's concept was well received, understood, and thought to be an important area of research. We consulted with the UK Centre for Tobacco and Alcohol Studies Smokers' Panel and the Elizabeth Blackwell Institute's Patient and Public Involvement Panel to develop interview schedules.

### 2.5 Research team and reflexivity

*GT* conducted the interviews, coding and analysis, and is a behavioural scientist and epidemiologist. *KS led on* coding and analysis of the interviews, and is a research assistant. *AS* had oversight of coding and analysis, and is a senior qualitative methodologist.

A working relationship was established with PWPs and stop smoking advisors prior to the interviews. There was no prior relationship with IAPT patients.

## 3 RESULTS

### 3.1 Participant profile

Eleven PWPs and six patients from IAPT, and six stop smoking advisors from smoking cessation services in the Midlands and South West regions of England took part in the study (Tables S5‐S7). Eleven PWPs who were invited to participate, agreed to participate. Eight IAPT patients were invited to participate, one did not reply, and one declined participation. Six stop smoking advisors who were invited to participate, agreed to participate.

PWPs worked across two large NHS trusts in England, and ranged from newly qualified to senior PWPs. Patients were seeking treatment for a variety of common mental illnesses (ie social phobia, anxiety and depression) and were daily smokers. Stop smoking advisors worked in a local authority supported and privately led smoking cessation service.

Below we present five themes, and 13 subthemes, with illustrative quotes (Table S8).

### 3.2 Theme 1: People with common mental illness use smoking to cope

Theme 1 characterizes patients’ motivations to smoke, and stop smoking advisors’ and PWPs’ perceptions of patients’ motivations to smoke. Theme 1 aligns with the COM‐B domain: ‘motivation’, and the TDF domain: ‘knowledge’.

#### 3.2.1 Smoking as a coping strategy

Smoking to cope appeared to be an ‘automatic’ and ‘reflective’ motive for smoking according to the COM‐B model. IAPT patients reported perceived psychological benefits from smoking, reported using smoking as a ‘crutch’, a comfort during stress, and that stress was a trigger for their ‘habit’ (Quotes 1‐2). Patient perceptions were echoed by stop smoking advisors who had perceived ‘knowledge’ that people with common mental illness used smoking to ‘psychologically cope’ (Quote 3). Overlapping with theme 2, PWPs also displayed perceived ‘knowledge’ that patients used smoking to cope, but classed it as an ‘unhelpful’ ‘safety behaviour’ that ‘artificially’ reduces unwanted symptoms like anxiety or stress (Quote 9).

#### 3.2.2 Smoking as a form of self‐harm

IAPT patients and PWPs described smoking as a form of self‐harm to replace ‘destructive’ behaviours like drinking alcohol, and as existing on a continuum from unhealthy lifestyle behaviours (ie overeating, smoking) to self‐injury (Quote 6). Smoking as a form of self‐harm was often described after reflecting on motivations for smoking (Quote 4). One participant described that she used self‐harm to ‘take the edge off’, and that self‐harm replaced smoking (Quote 5). Smoking as a form of self‐harm appears to act as both an ‘automatic’ and ‘reflective’ motivation for smoking (Table S8). Another patient reported the ‘reflective’ motivation of smoking as a form of self‐harm and that smoking was a ‘socially acceptable way of having (a) destructive coping mechanism’ (Quote 4).

### 3.3 Theme 2: Smoking as a vicious cycle

IAPT works predominately using a cognitive behavioural therapy (CBT) model. Theme 2 describes how patients experience a CBT cycle in the context of their tobacco addiction (ie the inter‐relationship between their thoughts, feelings, behaviours and physical sensations^31^), and how PWPs and stop smoking advisors perceive the cycle. Theme 2 aligns with the COM‐B domain: ‘motivation’, and TDF domains: ‘knowledge’ and ‘skills’.

#### 3.3.1 How IAPT patients experience the cycle

IAPT patients described how tobacco addiction related to their thoughts, feelings, behaviours, and physical sensations, and described how these processes interact. Patients’ experience of the cycle appeared to be both an ‘automatic’ and ‘reflective’ motivation for smoking. Patients explained that smoking caused feelings of ‘relief’, diminishing the physical craving, which led to feeling ‘calmer’, but also recognized that this process may be nicotine withdrawal (Quote 7).

#### 3.3.2 How PWPs and stop smoking advisors perceive the cycle

PWPs and stop smoking advisors displayed perceived ‘knowledge’ and ‘skills’ when describing tobacco addiction in terms of a CBT cycle. PWPs noted that the tobacco withdrawal cycle could ‘add to… anxiety symptoms because tobacco addiction… mimics symptoms of anxiety’ (Quotes 8‐9). Stop smoking advisors explained that smoking tobacco can lead smokers to ‘feel’ like they are ‘alleviating stress’, but that they will spend most of their day ‘craving’ tobacco and therefore feeling ‘stressed’ and ‘anxious’ (Quote 10).

#### 3.3.3 IAPT patient ‘buy‐in’

The interviewer (*GT*) presented research showing that stopping smoking is associated with mental health benefits and asked what participant's thoughts were about this finding. All IAPT patients responded positively to this message. Mapping on to COM‐B’s ‘reflective motivation’ domain, one IAPT patient mentioned that framing smoking cessation messaging in this way might motivate her to try and quit (Quote 11). Another patient hypothesized that quitting smoking could benefit physical *and* mental health, ‘quitting smoking is gonna make your physical health better, which I guess it would (then)… make your mental health better’ (Quote 12). *GT* further explained that researchers believe that mental health improves after quitting smoking because of breaking the tobacco addiction cycle, IAPT patients reflected on their own mental health and tobacco addiction in the context of this hypothesis (Quotes 13‐14), mapping on to the ‘reflective motivation’ domain.

### 3.4 Theme 3: IAPT as a natural infrastructure for offering smoking cessation treatment

Theme 3 describes intervention barriers and a negative ‘organisational culture’ towards smoking cessation treatment for people with common mental illness in smoking cessation services, and intervention facilitators and a positive ‘organisational culture’ towards smoking cessation treatment for people with common mental illness in IAPT. Theme 3 aligns with TDF domains: ‘knowledge’, ‘skills’, ‘beliefs about capabilities’, ‘optimism’, and ‘environmental context and resources'.

#### 3.4.1 Therapeutic pessimism and stigmatizing attitudes towards helping people with common mental illness to quit

In England people with common mental illness who seek help to quit are likely to receive treatment from smoking cessation services. Interviews with stop smoking advisors were conducted to learn about techniques that they use to support people with common mental health difficulties to quit smoking, we instead found potential environmental barriers. Stop smoking advisors perceived that they had the relevant ‘skills’ to support smoking cessation, but had uncertain ‘beliefs about (their) capabilities’ when treating smoking in this population, ‘in these patients, the motivation doesn't come from within… it doesn't last… they're less self‐motivated… so as a result it's much harder to carry them on the course’ (Quote 16). There was evidence that the ‘environmental context’ and ‘organisational culture’ in smoking cessation services was pessimistic and stigmatizing towards the ability of people with common mental illness to stop smoking. One stop smoking advisor inadvertently suggested that people with depression are somehow reprehensible and therefore cannot stop smoking (Quote 15). Stop smoking advisors reflected on the difficulty of treating people with common mental illness because they are ‘less self‐motivated’ and struggle with commitment (Quotes 16‐17). Another stop smoking advisor mentioned that smoking helps people with mental illness to ‘stop thinking about their problems’ (Quote 18).

#### 3.4.2 Stop smoking advisor acknowledgement that smoking cessation and psychological support may be complementary services

Stop smoking advisors displayed perceived ‘knowledge’ that people with common mental illness can often struggle with significant stress, that stop smoking advisors did not have the ‘skills’ to ‘deliver stress management’, and also indicated that they were not confident in providing advice about stress (Quotes 19‐20). Another stop smoking advisor commented on ‘environmental context and resources’ and mentioned that she had requested that their service offer stress management as part of smoking cessation treatment, but her request has not been implemented (Quote 19); potentially indicating another environmental ‘barrier’ to treating smoking cessation in people with common mental illness in stop smoking services.

#### 3.4.3 PWPs express perceived ability to provide behavioural support

PWPs referred to behavioural change models and techniques, and described how they usethese models clinically to motivate patients to make lifestyle changes when patients experience low motivation. PWPs showed ‘knowledge’ and ‘professional confidence’ in their understanding of depression and low motivation and how these symptoms can be managed in the context of smoking cessation treatment, ‘one of the main things… about depression is just that sense of demotivation, and getting people to recognize that's the voice of depression and that's what maintains the depression’ (Quote 21). PWPs expressed perceived ‘knowledge’ by describing models like COM‐B to assess patients’ desire to change behaviour; and that once the patients’ capability, opportunity and motivation are determined, PWPs then use this information to inform treatment planning (Quote 22). PWPs described their perceived ‘skills’ in using motivational interviewing and behavioural activation techniques to explore behaviour, encourage behaviour change and re‐engage patients with low motivation (Quote 23‐25). PWPs also expressed ‘beliefs about (their) capabilities’, such as ‘professional confidence’, and ‘perceived competence’. For example, one PWP stated ‘If (the patient) is not in a place where they can change, then we (try) to motivate them to get to that point’ (Quote 22). Another PWP noted that smoking cessation treatment in IAPT patients might be ‘harder’ but showed ‘optimism’ in how she could overcome this barrier clinically (Quote 24). In general, these findings indicate professional ‘optimism’ and that the ‘environmental context and resources’ are ‘facilitators’ to integrating smoking cessation treatment within IAPT.

#### 3.4.4 Integrating smoking cessation support into IAPT treatment

PWPs thought that integrating smoking cessation treatment into IAPT could complement IAPT’s ‘organisational culture/climate’, and that smoking cessation treatment could be logically positioned within the current IAPT treatment model. PWPs described their ‘knowledge’ and ‘skills’ in changing many types of behaviours as part of routine care, but noted that smoking cessation treatment appeared to be a gap in what IAPT offers, ‘we work on sleeping… eating… exercise… caffeine… the only thing we don't really touch is smoking’ (Quote 26). PWPs displayed ‘beliefs about (their) capabilities’ and ‘perceived competence’ in their ability to treat tobacco addiction in IAPT by comparing smoking to other ‘safety behaviours’ that they help patients to manage, ‘(smoking is) similar to many other kinds of safety behaviours… responses that people might have as something that feels better in the short term, but in the long term can make you feel worse’ (Quote 27). PWPs showed ‘professional confidence’ when describing how they could treat smoking in IAPT patients, ‘it's about identifying (smoking cessation) as a goal in your first session… I think using… the hot cross bun to be able to identify if smoking is a coping behaviour, or if smoking is related (to their mental health)’ (Quote 28). Displaying ‘optimism’ one PWP highlighted that smoking cessation treatment would ‘sit really nicely in the IAPT service’ (Quote 26).

### 3.5 Theme 4: Risk management

Theme 4 explores the concept of mental health risk and how this is managed across smoking cessation and IAPT services. Theme 4 aligns with TDF domains: ‘knowledge’, ‘skills’, ‘social/professional role and identity’, ‘beliefs about capabilities’, ‘optimism’ and ‘environmental context and resources'.

#### 3.5.1 Potential impact of psychological withdrawal symptoms

The interviewer (GT) noted that smoking cessation can cause tobacco withdrawal symptoms like low mood and anxiety, and asked how this could fit into a service that aims to help improve patient's mental health. PWPs accepted this concept and thought that the experience of tobacco withdrawal symptoms fits within IAPT’s treatment model. PWPs described their perceived ‘knowledge’ and ‘skills’ and described how this concept could fit into the ‘environmental context’, with ‘optimism’. PWPs appeared to be confident in ‘beliefs about (their) capabilities’, and the idea that delivering evidence‐based treatments that can sometimes make patients ‘feel worse before they feel better’ was part of their ‘professional role’ within IAPT (Quotes 29‐30). PWPs indicated that the ‘environmental context’ could facilitate smoking cessation treatment given potential negative psychological symptoms of tobacco withdrawal, ‘the end goal is… to get (patients) to feel better in the long‐term… and if they can do that whilst they're in our support I think that's probably better…’ (Quote 29).

#### 3.5.2 Risk management in the context of smoking cessation as an integrated treatment

Stop smoking advisors did not show ‘professional confidence’ when describing ‘beliefs about (their) capabilities’ in managing mental health risk. One advisor suggested that she would prefer to seek additional information and advice from GPs to enable her to ‘speak to’ patients with common mental illness and to mitigate potential mental health risk during smoking cessation treatment (Quote 31). PWPs raised the concept of risk, and described that assessing and managing patient risk was part of their ‘social/professional role and identity’ (Quote 32). PWPs were confident in their ‘skills’, ‘knowledge’ and ability to assess risk, demonstrating ‘perceived confidence’ and ‘professional confidence’ in ‘beliefs about (their) capabilities’. PWPs quickly applied their ‘professional role and identify’ when describing how they might manage risk related to smoking cessation treatment, and did so with ‘optimism’. PWPs indicated that ‘risk management’ was especially relevant to smoking cessation treatment, because if a patient feels that they need smoking to cope, or if they fail to stop smoking and feel worse as a result, the patient would be supported in IAPT if their mental health deteriorates, ‘if (patients) are feeling worse (mentally), then it's a safe place for them to tell us. We assess their risk every single session, we ask if they have any thoughts of suicide… self‐harm (and) check how risky they feel…’ (Quotes 32‐33). Risk management as part of IAPT’s routine procedures appeared to be a potential ‘facilitator’ in the ‘environmental context and resources’ TDF domain.

### 3.6 Theme 5: Intervention refinement and evaluation

PWPs explored training and service requirements to enable integration of smoking cessation treatment into IAPT services. Theme 5 aligns with TDF domains: ‘knowledge’, ‘skills’, ‘beliefs about capabilities’ and ‘environmental context and resources'.

#### 3.6.1 PWP training requirements

PWPs unanimously perceived that they had the ‘skills’, and showed ‘professional confidence’ in ‘beliefs about (their) capability’ to deliver smoking cessation treatment alongside usual IAPT care. PWPs identified gaps in their ‘knowledge’ and indicated that they would like training on the ‘evidence‐base’ of smoking and mental health, ‘I think we've got the skill‐base, it's just (the) research and evidence‐base’ (Quote 34). PWPs identified topics for training, like nicotine replacement (Quotes 35‐36), and identified the importance having ‘key points to drive home to patients’ about smoking and mental health (Quote 37).

#### 3.6.2 Messages for commissioners

PWPs displayed perceived ‘knowledge’ of the ‘task environment’ and identified various circumstances within IAPT’s ‘environmental context and resources’ which may act as barriers to integrating smoking cessation treatment within IAPT. PWPs emphasized the importance of careful messaging to IAPT patients ‘I don't want people to be put off accessing mental health services because they think that we're gonna jump in on telling them to stop smoking’ (Quote 38). PWPs highlighted the impact of austerity on IAPT, and that integrating smoking cessation treatment might require a change in treatment session duration, or a reduction in case‐load (Quotes 39‐40).

## 4 DISCUSSION

### 4.1 Summary

We aimed to understand patient experiences of comorbid smoking and common mental illness and views about treatments for tobacco addiction and common mental illness, including integrated treatment of both; health professional's knowledge and views of integrated treatment of tobacco addiction and common mental illness; and collect data to inform a potential smoking cessation intervention and training for integration into IAPT.

Similar to other studies^32^, ^33^ people with common mental illness reported perceived psychological benefits from smoking tobacco. Our study provided new insights into the idea that tobacco is used as a form of self‐harm. A common theme identified throughout the interviews was that tobacco addiction was described as a vicious cycle, or coined as an ‘unhelpful safety behaviour’. This study provides new understanding into how people with common mental illness can see how tobacco addiction is related to their thoughts, feelings, behaviours, and physical sensations, and how PWPs automatically integrate the tobacco withdrawal cycle into this CBT model. PWPs and patients recognized how the tobacco withdrawal cycle mimics common mental illness symptoms, how tobacco withdrawal may worsen mental health symptoms, and how breaking this cycle could benefit mental health. Stop smoking advisors displayed therapeutic pessimism and stigmatizing attitudes towards helping people with mental illness to quit, and did not display confidence in their ability to support people with common mental illness to quit smoking. PWPs displayed perceived confidence and optimism in their ability to help people with common mental illness to quit smoking, and believed that treating smoking in IAPT would fit well within the IAPT treatment model. Our study provides preliminary evidence that IAPT may be a suitable setting for offering smoking cessation treatment to patients who would like help to quit, and that IAPT may be more appropriately suited to offer smoking cessation treatment to patients with common mental illness than current smoking cessation services. Importantly, our findings mapped on to various COM‐B and TDF domains which will be useful for future intervention development and implementation.

### 4.2 Strengths and limitations

We sampled from two IAPT services in England, which may make findings transferable to other IAPT services. We only recruited stop smoking advisors from one service, therefore we are unsure about how transferable the findings from the smoking cessation service are we used a snowballing strategy for recruitment because PWP and stop smoking advisors have a high caseload, and recruitment via service managers overcame this. However, this recruitment method may introduce bias into our sample as service mangers potentially invited only motivated and receptive team members. It should also be noted that there was some variability in the depth of interviews conducted with IAPT patients; telephone interviews tended to be less reflective and more descriptive.

### 4.3 Comparison with existing literature

The data presented in this study are different compared with some studies of mental health professionals.^10^, ^21^, ^22^ Our study appears in indicate that PWPs have positive attitudes to implementing smoking cessation treatment into psychological services for people with common mental illness and that PWPs have a good understanding about how smoking could negatively impact on mental health and wellbeing.

Sheals and colleagues conducted a mixed‐methods systematic review and meta‐analysis of mental health professionals’ attitudes to treating smoking cessation in people with mental illness. Health professionals commonly reported that patients were not interested in quitting and that quitting smoking was too much for patients to take on. This review included professionals working predominately in services that implemented medically based treatment models, but did include a range of medical (eg psychiatrists, nurses) and ‘non‐medical’ professionals (eg psychologists, counsellors).^10^ A mixed‐methods study identified staff attitudes that acted as possible barriers to implementing ‘smoke‐free policy’ into in‐patient settings.^21^ Another survey of predominantly health‐care assistants, nurses and occupational therapists from across 25 UK inpatient units indicated that most staff felt that dealing with patient smoking was not their responsibility, and that smoking was an important coping mechanism for patients; these findings appeared to be consistent across medical and non‐medical staff.^34^ Smith conducted five focus groups conducted in English primary and secondary care services, including medical (eg psychiatrists, nurses), and non‐medical (eg PWPs, psychologists) staff^22^; findings indicated that staff believed that mental health patients were not motivated to stop smoking, or that their clinical role was not to ‘police smoking’. However, it is difficult to decipher differences between PWPs and other staff who took part in Smith's study.

In our study, it emerged that IAPT may be a more appropriate place to offer smoking cessation treatment than stop smoking services where people with common mental illness would usually be referred.^35^ We found that stop smoking advisors had stigmatized views about helping people with common mental illness to stop smoking. This is curious as their training involves specific modules about helping this population to stop smoking, and breaking down common myths about smoking cessation in this population.^18^ We cannot ascertain that these views are generalizable to other smoking cessation services; however, a survey of stop smoking advisors found similar attitudes.^36^ This finding does indicate a potential barrier for people with common mental illness when attempting to stop smoking via local stop smoking services; and signifies the relevance of PWPs in helping people with common mental illness to make lifestyle changes.

PWPs clearly understood that mental and physical health were inter‐related, and naturally accepted the idea of offering an integrated smoking cessation treatment for IAPT patients who were interested in receiving help to quit smoking. PWPs accepted the notion that stopping smoking may induce psychological withdrawal symptoms and did not see this as a problem in the context of usual IAPT care. PWPs noted that often patients ‘get worse before they get better’. PWPs described how risk is routinely assessed and managed in IAPT, and how IAPT’s risk protocol could be used to support provision of smoking cessation treatment. There was evidence that PWPs might sometimes use health psychology models that are not supported by evidence. For example, one PWP mentioned that using the ‘Stages of Change’ model might be a useful way to explore a patient's thoughts about changing their smoking behaviour. Findings from a systematic review do not support the view that finding out what ‘stage of change’ a patient is at before offering help to stop smoking is useful.^37^ Equally, there was evidence that PWPs also use evidence‐based models, like COM‐B to support behaviour change.

PWPs noted that if smoking cessation treatment was to be offered in IAPT, service leads should carefully frame this as patients could be deterred from mental health treatment if smoking cessation treatment was perceived as mandatory. PWPs also noted that if smoking cessation treatment were to be integrated into IAPT that longer treatment sessions, or smaller case‐loads might be necessary. Given that smoking cessation interventions are among the most cost‐effective health care interventions available^38^ and that smoking cessation is linked to considerable mental health benefits,^11^ offering smoking cessation as part of routine mental health care seems sensible from a patient‐, commissioning‐ and NHS‐perspective.

### 4.4 Implications for research and/or practice

IAPT patients and PWPs welcomed the idea of smoking cessation as an integrated treatment to improve mental health. They were able to reflect on their experiences and identify examples of when tobacco withdrawal has mimicked anxiety or depression symptoms, and how this relates to their mental illness. PWPs were able to link tobacco withdrawal to a CBT vicious cycle in which thoughts, feelings, behaviours, and physical sensations are interrelated and can maintain unhealthy coping mechanisms. PWPs identified the clinical implications of using this cycle as a psychoeducational tool during IAPT treatment sessions.

In England, people with common mental illness who seek help to quit are usually referred/self‐referred to smoking cessation services. Interviews with stop smoking advisors were conducted to learn about behavioural techniques that they use to support people with common mental illness to stop smoking, but an unexpected finding was that stop smoking advisors had pessimistic and stigmatizing attitudes towards helping people with common mental illness to quit smoking, and lacked confidence in their ability to treat smoking cessation in this population. It is important that smoking cessation services complete the ‘smoking and mental health’ module provided by NCSCT.^39^ Furthermore, these findings seem to suggest that integrated smoking cessation services for people with common mental illness may be more appropriate.

It might be possible to integrate smoking cessation treatment into IAPT, using a modified version of NCSCT’s standard treatment programme for smoking cessation,^18^ whereby PWPs focus on addressing the relationships between smoking, the withdrawal cycle and links to mental health, using a CBT model.^31^ Integration of smoking cessation treatment into IAPT services should be tested in a pilot and feasibility trial. Our findings mapped on to COM‐B and TDF domains which will be useful to consider in potential intervention development and implementation.

## 5 CONCLUSION

IAPT PWPs and patients accept evidence that smoking tobacco may harm mental health, quitting might benefit mental health, and welcomed the idea of smoking cessation as an integrated treatment within IAPT to improve mental health and overall wellbeing. PWPs held positive attitudes towards smoking cessation treatment for people with common mental illness and displayed confidence in helping this population to stop smoking in the face of mental illness and low motivation. Our study provides preliminary evidence that IAPT may be a more suitable setting for offering smoking cessation treatment to patients who would like help to quit, than current smoking cessation services, however, a reduction in PWP case‐load may be required. Our findings mapped on to COM‐B and TDF domains which will be useful to consider in potential intervention development and implementation in a future pilot and feasibility trial.

## CONFLICT OF INTEREST

Dr Alison Heawood (nee Shaw) and Prof David Kessler have no conflicts of interest. Dr Gemma Taylor and Prof Marcus Munafò have previously received funding from Pfizer, who manufacture smoking cessation products, for research unrelated to this study. Prof Paul Aveyard led a trial funded by the NIHR and GlaxoSmithKline donated nicotine patches to the NHS in support of the trial.

## AUTHORS' CONTRIBUTION

Dr Gemma Taylor lead on study conceptualization, methodology, analysis, investigation, writing and editing the manuscript, administration, and funding acquisition, contributed to data curation and supervision of analysis. Ms Katherine Sawyer led on data curation, data analysis, and contributed to investigation, writing and editing the manuscript and project administration. Prof David Kessler contributed to study conceptualization, methodology, analysis, investigation, writing and editing the manuscript, and funding acquisition. Prof Marcus Munafò supervised study conceptualization, investigation and funding acquisition, and contributed to study methodology, analysis, writing and editing the manuscript and project administration. Prof Paul Aveyard supervised study conceptualization, analysis, investigation, writing, editing and funding acquisition, and contributed to methodology and project administration. Dr Alison Heawood led on supervision of methodology, formal analysis, investigation, writing and editing the manuscript, and contributed to study conceptualization, project administration and funding acquisition.

## Funding information

Dr Gemma Taylor and Ms Katherine Sawyer are funded by Cancer Research UK (Population Researcher Postdoctoral Fellowship award (C56067/A21330). Dr Alison Heawood (nee Shaw) is funded through various NIHR grants. The MRC Integrative Epidemiology Unit at the University of Bristol is supported by the Medical Research Council and the University of Bristol (MC_UU_00011/7). Prof David Kessler is funded by The Centre for Primary Care at the University of Bristol. Prof Paul Aveyard is funded by NIHR, CRUK, Wellcome Trust, BMA Foundation, and MRC, and the NIHR Oxford Biomedical Research Centre and Applied Research Centre.

## ETHICAL APPROVAL

Ethics approval for this study was received from the NHS research ethics committee on 13 July 2017 (Reference 17/WM/0251).

## Supporting information

Supplementary Material

## Data Availability

Anonymized transcript data are available for free via application to the University of Bath Research Data Archive (https://doi.org/10.15125/BATH‐00921).
