# Supplementary material for: Views about integrating smoking cessation treatment within psychological services for patients with common mental illness: A multi‐perspective qualitative study
Source: Health Expect. 2020 Dec 23;24(2):411–20. doi: 10.1111/hex.13182 (PMC8077097; doi:10.1111/hex.13182)
Supplement: Supplementary file 1 — Supplementary Material [file HEX-24-411-s001.docx]

**ONLINE SUPPLEMENT**

**TABLE OF CONTENTS**

[Table 1 Semi-structured interview schedule for IAPT patients 2](#_Toc43299152)

[Table 2 Semi-structured interview schedule for IAPT psychological wellbeing practitioners (PWPs) 3](#_Toc43299153)

[Table 3 Semi-structured interview schedule for stop smoking service advisors 4](#_Toc43299154)

[Table 4 Examples of text that were coded in more than one way, both inductively and deductively 5](#_Toc43299155)

[Table 5 IAPT patient characteristics 6](#_Toc43299156)

[Table 6 IAPT psychological wellbeing practitioner (PWP) characteristics 6](#_Toc43299157)

[Table 7 Stop smoking advisor characteristics 6](#_Toc43299158)

[Table 8 Quotes from IAPT psychological wellbeing practitioners (PWPs) and patients, and stop smoking advisors 7](#_Toc43299159)

**Table 1 Semi-structured interview schedule for IAPT patients**

| **Gain an understanding of patients’ experience of smoking and quitting to contextualise later questions.**   - Do you remember the reasons why you started smoking? What were they? - Have you ever tried to quit smoking? - What motivated you to quit? - What helped you in quitting? - What made it difficult to quit/stay quit? - If you’ve never tried to quit, do you mind me asking the reasons why you’ve not ever tried?   **Perspectives about treatments for smoking cessation.**   - Have you ever used prescription or over-the-counter medication, or e-cigs to help you quit smoking? - Have you ever been offered behavioural support? - What do you think the benefits or risks of using medications/e-cigs are?   **Experience of comorbid smoking and mental health.**   - Can you explain the ways that your mental health and smoking are related?   - Can you describe a time/example? - Can you explain the ways in which smoking might be good or bad for mental health? - Explain how the withdrawal cycle can mimic mental health symptoms.   - How does this relate to your own mental health?   **Perspectives about parallel treatments of smoking cessation and depression.**   - Do you see any issues or benefits of delivering a smoking cessation intervention as an adjunct to usual depression treatment? - What would be the best way to help someone quit smoking using medications/e-cigs while they are receiving depression treatment? - What would be good and less good about helping someone quit smoking while they were receiving depression treatment? - How do you think smoking cessation treatment may be best introduced into therapy settings?   **How to optimise recruitment, retention, treatment adherence?**   - What have been the good/less good things about the care you received in IAPT?   **Is there anything else that you would like to mention?** |
| --- |

**Table 2 Semi-structured interview schedule for IAPT psychological wellbeing practitioners (PWPs)**

| **Explore PWP skills**   - In what ways do you help patients change unhealthy lifestyle behaviours? - What types of therapy/techniques do you use in IAPT for treating depression? - Which interventions do you offer? - Do you sometime address lifestyle behaviours in your treatment plan? What prompts you to do so and what approach do you take. - Do you do any other addiction work?   **Explore professional identity**   - Do you feel that part of your role is to help people change lifestyle behaviours, like smoking?   **Explore PWP knowledge**   - Can you explain the ways that smoking might be good or bad for mental health? - How do you think that stopping smoking can affect mental health? - Does it matter that a person tries to stop smoking when they are depressed? - Do you ever give advice about using smoking cessation medications? If so what? - Are there any benefits or risks of smoking cessation medications?   **Perspectives about the smoking cessation intervention**   - Do you see any issues/benefits of delivering smoking cessation intervention as an adjunct to usual IAPT care? - How do you feel about your patients trying to quit smoking while their using IAPT services? - What would motivate you to deliver the smoking intervention?   **Integration into IAPT treatment**   - How do you feel this intervention would best fit into your practice? - Do you notice any over-lapping techniques/theories between your practice and the behavioural stop smoking treatment? - How do you think it’s best to integrate the stop smoking treatment? - When to you think it would be best to start the SS treatment during an IAPT therapy session? - Do you see any barriers/facilitators to introducing SS treatment in to IAPT treatment? - Are there any service level barriers/ facilitators to its delivery?   **Retention**   - What are some of the main reasons patients drop out of /remain in treatment? - Do you think there are any solutions to this? - Do you have any suggestions about how to retain participants in the treatment and study?   **Therapist training needs**   - How could we help you in delivering the intervention? - What training do you think you need?   **Is there anything else that you would like to mention?** |
| --- |

**Table 3 Semi-structured interview schedule for stop smoking service advisors**

| **Elicit views about co-morbid smoking and mental health**   - Do you think that smoking and mental health are related? If so, in what ways? - Can you tell me about how smoking might be good or bad for mental health? - What are the current barriers and facilitators to retaining patients in the Stop Smoking Services? - Thinking about your patients with low mood/depression – can you tell me about the reasons that they drop out/remain in treatment? - Do you think there are any solutions to this?   **Talking to patients about the relationship between their mental health and smoking**   - How would you go about reassuring a patient w/low mood about psychological withdrawal symptoms? - How would you go about reassuring a patient who uses smoking to cope with stress and mood? What would you say? - How do you target beliefs that smoking is mood enhancer? What would you say? - What skills do you drawn upon when talking to patients about the relationship between smoking and mental health?   **Intervention manual behavioural components**   - Which of these items are most/least useful? And In people who rely heavily on tobacco for mood enhancement, pleasure, stress? - How long does each component take you? - Do you combine any techniques together? - What feedback could we use instead of CO monitoring? - How do you use the Heaviness of Smoking Index to inform your practice/prescribing?   **Intervention manual pharmacological components**   - If someone reports depression/mood disorders, what’s the standard medication procedure? - What’s the standard medication procedure for high dependency patients? - How do you address rumors around varenicline and mental health symptoms?   **Trial specific**   - Do you see any issues or benefits of delivering a smoking cessation intervention as adjunct to usual depression treatment? - Do you have any suggestions about how to motivate participants to stay in treatment?   **Is there anything else that you would like to mention?** |
| --- |

**Table 4 Examples of text that were coded in more than one way, both inductively and deductively**

| Deductive: PWP Knowledge/skills  Inductive: PWPs express their ability provide behavioural support  *“If they wanted to change it* (smoking)… *there has to be a desire to change it, so in the assessment we’re checking three factors, so it’s called COMBI … so it’s their, um, capability to change, the opportunity they’ve got to change and the motivation they’ve got to change their behaviour, so those three factors, if they’re keen to change it, then we would look at integrating it and look at making those changes. If they’re not in a place where they can change, then we look at kind of trying to motivate them to get to that point. So they have to be ready to change, ’cause it’s got to be their choice, so they may not be in a place where they’re able to do it, and actually, we may just be encouraging them, supporting them to do the basics like get out of bed, have a shower, that kind of thing, if they’re particularly low. Um, if they are motivated and they’re really keen to make some change and actually we’ve done the COMBI assessment and checked on their capabilities, opportunities and motivation and they’re ready to make that change, we then push them a little bit further.”*   - - Female PWP, aged 30 years, 4 years’ experience   *“I think it would be harder because of their low motivation and I think that’s why exploring the beliefs around it might be helpful and um exploring with them their readiness for change, so in terms of motivational – their motivation, where they are on that… so when somebody is thinking about making a change, they go through different stages. So, off the top of my head, you go through a pre-contemplation, a contemplation and then like a part where you are then ready to change, and if you’re in this part here, this stuff would be really good because you move them from this kind of precontemplation to action.”*   - - Female PWP, aged 28 years, 4 years’ experience   Deductive: Using smoking as a coping mechanism  Inductive: Smoking as a vicious cycle  *“If I have one* (cigarette) *it’s sort of a bit of a relief, not only just maybe five, ten minutes to myself to think it’s more that my body is getting what it’s craving as well and it just eases off and it’s a calmer feeling anyway but that’s probably me just not recognising the fact that I don’t get the nicotine or something”*   - - Male IAPT patient, aged 32 years, generalised anxiety disorder   *“Well it just makes me feel less anxious. Makes me feel a little bit calmer but then obviously you know you’re craving the next cigarette; you know you’re craving the next hit to keep you feeling calm.”*  *-* Female IAPT patient, aged 44 years, mixed anxiety and depressive disorder |
| --- |

**Table 5 IAPT patient characteristics**

| **ID** | **Age** | **Primary diagnosis** | **PHQ-9 score** | **GAD-7 score** | **Sex** |
| --- | --- | --- | --- | --- | --- |
| 1 | 44 | Mixed anxiety and depressive disorder | 14 | 14 | Female |
| 2 | 54 | Depressive episode | 20 | 13 | Female |
| 3 | 26 | Social phobias | 15 | 15 | Female |
| 4 | 60 | Mixed anxiety and depressive disorder | 17 | 19 | Female |
| 5 | 32 | Generalised anxiety disorder | 13 | 15 | Male |
| 6 | 34 | Social anxiety | 9 | 8 | Male |

**Table 6 IAPT psychological wellbeing practitioner (PWP) characteristics**

| **ID** | **Age** | **Years working as PWP** | **Site** | **Sex** |
| --- | --- | --- | --- | --- |
| 01 | 27 | 1 | 1 | Female |
| 02 | 24 | 2 | 1 | Female |
| 03 | 30 | 4 | 1 | Female |
| 04 | 28 | 2 | 1 | Female |
| 05 | 28 | 3 | 1 | Male |
| 06 | 35 | 4 | 1 | Male |
| 01 | 32 | 3 | 2 | Female |
| 02 | 28 | 1 | 2 | Female |
| 03 | 30 | 1 | 2 | Female |
| 04 | 28 | 4 | 2 | Female |
| 05 | 27 | 2 | 2 | Female |

**Table 7 Stop smoking advisor characteristics**

| **ID** | **Age** | **Years working as a stop smoking advisor** | **Site** | **Sex** |
| --- | --- | --- | --- | --- |
| 01 | 28 | 2 | 1 | Female |
| 02 | 35 | 4 | 1 | Male |
| 03 | 34 | 2 | 1 | Female |
| 04 | 36 | 1 | 1 | Female |
| 05 | 33 | 2 | 1 | Female |
| 06 | 39 | 2 | 1 | Male |

**Table 8 Quotes from IAPT psychological wellbeing practitioners (PWPs) and patients, and stop smoking advisors**

| **Theme** | **Subtheme** | **COM-B domain** | **TDF domain** | **Quote(s)** |
| --- | --- | --- | --- | --- |
| Theme 1: People with common mental illness use smoking to cope | Smoking as a coping strategy | Automatic motivation  Reflective motivation | Knowledge | Quote 1: “*I did a good nine months using* (smoking cessation medicine) *but then something quite traumatic happened … I just literally used smoking as a crutch really to kind of deal with it. It* (smoking) *was just kind of like an old habit that I fell back into because I felt like smoking had got me through other situations in life.”*   - Female IAPT patient, aged 44-years, mixed anxiety and depressive disorder   *Quote 2: “*(I smoke) *particularly if there’s quite a short deadline at work or something or if I get into an argument with somebody like the missus or something like that – an extreme argument not just the normal ones and then that might trigger it* (smoking)*.”*   - Male IAPT patient, aged 32-years, generalised anxiety disorder   Quote 3: “Cigarettes is one of the common ways psychologically (they) cope, they (people with common mental illness) think it’s a stress release.”   - Male stop smoking advisor, aged 39-years, 2-years’ experience |
|  | Smoking as a form of self-harm | Automatic motivation  Reflective motivation | Knowledge | Quote 4*: “…it* (smoking) *might be kind of like a weird form of like self-harm in a way as well ‘cause when I… initially started smoking for maybe like a month whether I was drinking or not and I realised that that was also kind of like a coping mechanism but it’s very self-destructive and I realised there was no way that I was gonna be able to carry on doing that and stay in university and then I guess I kind of started smoking when I was going out. I was like ‘Okay well I’ll just stop drinking because that’s not something I’m gonna be able to like maintain’, but like smoking is a more socially acceptable way of having that kind of like destructive coping mechanism…”*   - Female IAPT patient, aged 26-years, social phobia   Quote 5: *“I was just sad all the time, I just wanted to have a cigarette because I thought there was nothing in life for me. I have done self-harming to myself as well … which does take the edge off so if I’m self-harming myself I won’t have a cigarette.”*   - Female IAPT patient, aged 60-years, mixed anxiety and depressive disorder   Quote 6: *“… if we were to think about … kind of self-harm and self-injury, and there’s quite a big scale of that, so self-harm starting with maybe, um, under-exercising or overeating or smoking, and that then going up to kind of self-injury. So, I suppose, when we think about if people are smoking, are they smoking to cope in some way with difficult emotions or what’s going on? Um, so is it as easy as we just start smoking, or is there more going on there that would need to be replaced or worked on in some way, um, I think, maybe. Um, and is it just a bit of a break from life, you know, what... what is it they’re... obviously, there’s addiction, but is there something else, a bit more, that’s... um, needs to be thought about, in a way...”*   - Female PWP, aged 27-years, 1-year experience |
| Theme 2: Smoking as a vicious cycle | How IAPT patients experience the cycle | Automatic motivation  Reflective motivation |  | Quote 7: *“Well it just makes me feel less anxious. Makes me feel a little bit calmer but then obviously you know you’re craving the next cigarette; you know you’re craving the next hit to keep you feeling calm.”*   - Female IAPT patient, aged 44-years, mixed anxiety and depressive disorder |
|  | How PWPs and stop smoking advisors perceive the cycle |  | Knowledge  Skills | Quote 8*: “If they’re already having physical problems they’re already internally focusing on their breathing, or palpitations…then smoking it can then add to the* (anxiety) *symptoms… because it* (tobacco addiction) *increases your palpitations and mimics the symptoms of anxiety… It’s definitely what we classify as an unhelpful behaviour.”*   - Female PWP, aged 28-years, 1-years’ experience   Quote 9: *“I do think that smoking can be used as an um safety behaviour particularly in anxiety so people with tend to use that as a way to reduce their anxiety… they’re artificially bringing their anxiety down which is unhelpful. So, I guess in some ways understanding the habit of smoking and getting them to reduce that in the situation with anxiety could be helpful as well. Also, I think in terms of fitting it in and around with interventions that work with depression* (like behavioural activation) *I think that would probably work quite well.”*   - Female PWP, aged 28-years, 4-years’ experience   Quote 10: *“Firstly, you tend to explain to the client, saying ‘This is what happens, you may feel you’re alleviating stress...you spend more time as a percentage of your day craving, and when you’re craving the usual association is that you are stressed, because you tend to get quite anxious as well’. If you plotted that on a graph you have tiny spikes of where you have a cigarette which is the alleviation, but the time that gives you, the area under the curve if you like, reduced anxiety and all the rest of it, it’s not that high. It spikes up quickly and spikes back down quite quickly, then in another hour you need to have another cigarette. Well, in that hour in-between the two cigarettes you could probably say 45 minutes of that has been, ‘Okay, I’m starting to get a bit itchy, I need to go and have a cigarette.’ So just to explain that to the clients, and some the physiology behind it can help.”*   - Male stop smoking advisor, aged 35-years, 4-years’ experience |
|  | IAPT patient ‘buy-in’ | Reflective motivation |  | Quote 11*: “Stopping smoking as a treatment for mental health would make me more likely to want to do it* (quit smoking). *I think if it was framed more as like quitting smoking can actually help your mental health, not like they just wanna like shoe horn it in ‘cause that’s the kind of idea that I kind of had initially that it was like ‘Okay this is the population that we know smoke a lot, we want people to stop smoking so let’s kind of like shoe horn the stop smoking in with the therapy to try and cut down the general population.’”*   - Female IAPT patient, aged 26-years, social phobia   Quote 12: *“I mean quitting smoking is gonna make your physical health better, which I guess it would probably make your mental health better”*   - Female IAPT patient, aged 44-years, mixed anxiety and depressive disorder   Quote 13: *“Maybe the withdrawal symptoms are causing my anxiety sometimes.”*   - Male IAPT patient, aged 32-years, generalised anxiety disorder   Quote 14: *“Yeah so when I’m clear of it* (tobacco addiction), *like clear of wanting one, out of the habit of going to one and it’s like been a lot of weeks maybe a month or two down the line, I do notice, yeah I’m a lot calmer and a lot happier actually. I have noticed that in the past when I’ve given up for a considerable period.”*   - Male IAPT patient, aged 32-years, generalised anxiety disorder |
| Theme 3: IAPT as a natural infrastructure for offering smoking cessation treatment | Therapeutic pessimism and stigmatising attitudes towards helping people with common mental illness to quit |  | Knowledge  Skills  Beliefs about capabilities  Environmental context and resources | Quote 15: *“Let’s say somebody has depression serious severe depression. They’re not going out, they’re not eating, their whole life has stopped, and they’re smoking. They just like to sit and smoke, and showing severe depression, so I feel that bringing them back to life will take a good amount of effort, however the effort to quit smoking may be too much effort in their life”*   - Female stop smoking advisor, aged 28-years, 2-years’ experience   Quote 16: *“More often than not we sign these people* (people with common mental difficulties) *up anyway, even if they chronically relapse and may come back into the service. The support we provide behaviourally is based around motivational interviewing and trying to get them to provide the motivation. Whereas in these patients, the motivation doesn’t come from within…it doesn’t last… they’re less self-motivated, and so as a result it’s much harder to carry them on the course.”*   - Male stop smoking advisor, aged 35-years, 4-years’ experience   Quote 17*: “Their* (people with common mental difficulties) *biggest problem is commitment. And a lot of things that actually become into depression and anxiety actually starts at the beginning, when they start to fail with commitments: things like, ‘Don’t worry, I’ll do that tomorrow’; but that never happens.”*   - Male stop smoking advisor, aged 39-years, 2-years’ experience   Quote 18: *“They smoke ‘cause of enjoyment. It’s to stop them from thinking about their problems. That’s why they smoke more than people who don’t have mental illness. That’s why they’re really, really difficult to work with.*   - Female stop smoking advisor, aged 34-years, 2-years’ experience |
|  | Stop smoking advisor acknowledgement that smoking cessation and psychological support may be complementary services |  | Knowledge  Skills  Beliefs about capabilities  Environmental context and resources | Quote 19: *“…nearly everybody has stress in their life, and we say we will just focus on the points they mostly find really hard to quit, like some people say, ‘Oh, I find it really hard when my children are driving me crazy,’ we say, ‘We will focus on that part, and you will develop your own coping strategy, and how you can distract yourself every time you feel that stress.’ However, it’s really difficult because we’re not trained to give stress management, so most of the people come and they start to talk about what stresses them in their life. We have to stop that part and say, ‘Okay, let’s talk about how you will cope to not smoke in those situations,’ so it’s really hard to differentiate it… for a long time I’ve been saying we should just do the stress management (when delivering smoking cessation treatment to patients with common mental illness).”*   - Female stop smoking advisor, aged 28-years, 2-years’ experience   Quote 20: *“I think on the behavioural support side because I’m not a trained psychologist that sometimes it is just providing support that’s based on my own personal beliefs like it’s nothing that’s scientific… they’ll say their thing and I try to not put too much of what, I think they should do because I’m not trained in any of that. I like them to try to come up with their one… If they are nervous about their situation I like them to come up with their own coping mechanism and I’ll discuss how they can implement it but I don’t want to tell them, ‘Oh I think you should do this,’ and this and this because I’m probably not right.”*   - Female stop smoking advisor, aged 36-years, 1-years’ experience |
|  | PWPs express perceived ability to provide behavioural support |  | Knowledge  Skills  Beliefs about capabilities  Optimism  Environmental context and resources | Quote 21*: “Somebody I spoke to the other day said, ‘well I, I did quit but since I’ve started feeling more stressed again, I’ve started smoking again’. So (s/he) might think um, ‘I’m failing at this - I’ve started smoking again’, and that might feed into more negative cognitions they might have about themselves… and equally one of the main things um about depression is just that sense of demotivation, and getting people to recognise that’s the voice of depression and that’s what maintains the depression.”*   - Female PWP, aged 27-years, 2-years’ experience   Quote 22: *“If they wanted to change it* (smoking)… *there has to be a desire to change it, so in the assessment we’re checking three factors: their capability to change, the opportunity they’ve got to change and the motivation they’ve got to change their behaviour. So, if they’re keen to change it, then we would look at integrating it and look at making those changes. If they’re not in a place where they can change, then we look at kind of trying to motivate them to get to that point.”*   - Female PWP, aged 30-years, 4-years’ experience   Quote 23: *“Um, so with obviously motivation um, we try to sort remind people of the pros and cons of change so that okay um, what do you – what’s wrong with your current situation. Do you want to make a change and what’s that change worth and er sometimes that can make people re-engage.”*   - Female PWP, aged 32-years, 3-years’ experience   Quote 24: *“I think (smoking) would be harder because of their low motivation and I think that’s why exploring the beliefs around (smoking) might be helpful and um exploring with them their readiness for change, so in terms of their motivation, when somebody is thinking about making a change, they go through different stages, a pre-contemplation, a contemplation and then a stage where they’re ready to change, and if they’re ready to change it* (smoking cessation intervention) *would be really good because you move them from pre-contemplation to action.”*   - Female PWP, aged 28-years, 4-years’ experience   Quote 25: *“if they have low mood, one of the techniques we look at is called behavioural activation, and we look at managing or changing their behaviours and increasing the positive behaviours to get more of a balance... So, it depends what they want to work on, what their goals are for treatment, but it can be worked on and integrated into the therapy if necessary, because if their lifestyle factors are impacting their mood and they aren’t eating properly, or exercising or they are kind of drinking a lot to manage, (we) have to adapt that behaviour in order to move forward, so it definitely can be integrated in.”*   - Female PWP, aged 30-years, 4-years’ experience |
|  | Integrating smoking cessation support into IAPT treatment |  | Knowledge  Skills  Beliefs about capabilities  Optimism  Environmental context and resources | Quote 26: *“I think it’s a really good idea because we work on sleeping. We work on eating. We work on exercise. We work on caffeine. We work on all the elements of somebody’s wellbeing and the only thing we don’t really touch is smoking, which is – we even work on alcohol use so to have treatment with us, you have to be below the alcohol limits. So actually, we look at a lot of these different things to treat people. Smoking seems to be the only one we don’t really touch, so that’s – I think it would sit really nicely in the IAPT service.”*   - Female PWP, aged 28-years, 1-years’ experience   Quote 27*: “So, um, it, it’s often I suppose similar to many other kinds of safety behaviours. We term safety behaviours, or responses that people might have as something that feels better in the short term, but in the long term can make you feel worse physically but also it can fuel more negative cognitions.”*   - Female PWP, aged 27-years, 2-years’ experience   Quote 28: *“Thinking about how that applies I guess to an intervention around smoking, it’s about identifying (smoking cessation) as a goal in your first session. So, we have a treatment planning session, where we plan treatment with (patients) collaboratively, so I think using things like the hot cross bun to be able to identify if smoking is a coping behaviour, or if smoking is related (to mental health) in any way, or if smoking is just something that they do, um, and then using that to sensitively question well is it something you want to look at or a goal that you want to think about.”*   - Female PWP, aged 28-years, 4-years’ experience |
| Theme 4: Risk management | Potential impact of psychological withdrawal symptoms |  | Knowledge  Skills  Social/professional role and identity  Beliefs about capabilities  Optimism  Environmental context and resources | Quote 29*: “As long as* (patients) *are aware that they will potentially feel worse and that they will have those withdrawals and it will be difficult but we’re here to support them. The key part is making them aware and then it’s their choice what they do. I don’t think there’s a problem with us doing that to someone or encouraging someone to* (quit smoking) *when they’re in mental health support. Because… the end goal is still to get them to feel better in the long-term… and if they can do that whilst they’re in our support I think that’s probably better…”*   - Female PWP, aged 30-years, 4-years’ experience   Quote 30: *“We are telling people to make changes and sometimes those changes can be quite significant and, quite distressing for the patient… sometimes people do feel worse before they feel better. So, we always tell people at the beginning of the treatment, that this is not going to be easy. That this is um evidence-based, it has good outcomes the treatments that we offer but… you might feel worse in the beginning before the treatments start to take effect.”*   - Female PWP, aged 32-years, 3-years’ experience |
|  | Risk management in the context of smoking cessation as an integrated treatment |  | Knowledge  Skills  Social/professional role and identity  Beliefs about capabilities  Optimism  Environmental context and resources | Quote 31: *“I think (patients with mental illness) should (provide) a number of a doctor… to be sure that (the patient) is on medication (so) you see that something happens … or (if they are) reacting differently than someone who don’t have any mental illness. (So that I can)… call … the doctor and ask exactly what’s happened and how to speak to this client.”*   - Female stop smoking advisor, aged 34-years, 2-years’ experience   Quote 32: “*if* (patients) *are feeling worse* (mentally)*, then it’s a safe place for them to tell us. We assess their risk every single session, we ask if they have any thoughts of suicide, any action taken, any plans to end their life; we look at thoughts of self-harm, any action taken, that kind of stuff, so essentially check how risky they feel. Um, thoughts are normal and we kind of normalise that to the patient. We say, ‘You know, if you’re standing on the train platform, um, you might have thoughts of “What if I jumped in front of that train?” Doesn’t mean you’re gonna act on it. You know, nine out of ten people have had that thought. I’ve had that thought.”*   - Female PWP, aged 30-years, 4-years’ experience   Quote 33: *“…we check in each appointment… because we know that things can change. Either if somebody is, somebody’s mood is worse. Somebody’s anxiety increases, um and that’s something that we might need to revisit. I’ve worked with people before who might initially at assessment have seemed okay, but when you get through for their appointment, or over the course of therapy… they feel worse, and maybe they start self-harming again, or having thoughts of suicide… because you’ve got a chance to revisit* (risk) *each time, then you might need to change treatment direction. You might say we need to send you to the adult mental health team… or maybe we need to think about high intensity therapy”*   - Female PWP, aged 27-years, 2-years’ experience |
| Theme 5: Intervention refinement and evaluation | PWP training requirements |  | Knowledge  Skills  Beliefs about capabilities | Quote 34*: “I think we’ve got the skill base, it’s just that sort of research and evidence base.”*   - Female PWP, aged 32-years, 3-years’ experience   Quote 35: *“I guess the effects of the different medications, patches and so on and maybe the differences between e-cigarettes… more information about those things so that we…know that we’re giving people facts. We’re an evidence-based service…”*   - Female PWP, aged 32-years, 3-years’ experience   Quote 36*: “*(I would like) *to know about physiology of um withdrawal. Are they more or less likely to get shortness of breath? Or do their airways as they get unblocked… do we encourage them to exercise at the same time, or we would focus on just smoking cessation and then start an exercise.”*   - Female PWP, aged 32-years, 3-years’ experience   Quote 37: “*Statistics about relationships between depression and anxiety and smoking. The key studies. The key points to take home, to drive home to patients would be really important… to know the effect on different disorders.”*   - Female PWP, aged 28-years, 1-years’ experience |
|  | Messages for commissioners |  | Knowledge  Environmental context and resources | Quote 38: *“I don’t want people to be put off accessing mental health services because they think that we’re gonna jump in on telling them to stop smoking and that sort of thing, um, which I don’t think it would be that anyway, but just being conscious of that, I suppose…”*   - Female PWP, aged 27-years, 1-years’ experience   Quote 39*: “One big problem that we have is sort um too many people in not the right step of the Step Care model because of cuts made in the psychological services and complex needs services, so – which means that we see a lot of complex patients which actually this kind of telephone guided self-help is not necessarily suitable for so then we might need to have longer session or more sessions or treat them for longer or um, it might get more complex so um, that’s a major um barrier.*”   - Female PWP, aged 32-years, 3-years’ experience   Quote 40*: “Um, maybe if, I’m not fully sure, the only one would be maybe if we’re offering longer appointments or having treatment for a bit longer, it maybe that we aren’t then seeing as many people as we would before because we’re holding onto those people for a bit longer so that could maybe implement the service in terms of numbers of people accessing treatment.”*   - Female PWP, aged 30-years, 1-years’ experience |
